# Mastectomy With Sentinel Lymph Node Biopsy Under Regional Anesthesia in a Patient With a History of Multiple Anaphylactic Episodes

**DOI:** 10.7759/cureus.83572

**Published:** 2025-05-06

**Authors:** Kateryna Samalyuk, Margarida Alves, Céline Ferreira, Margarida Gil Pereira

**Affiliations:** 1 Anesthesiology, Unidade Local de Saúde de Coimbra, Coimbra, PRT; 2 Allergy and Clinical Immunology, Unidade Local de Saúde de Coimbra, Coimbra, PRT

**Keywords:** allergy and anaphylaxis, analgesia cancer-related breast surgery, awake breast surgery, perioperative case report, thoracic epidural anesthesia, ultrasound guided regional anesthesia

## Abstract

We describe the case of a 55-year-old woman proposed for an elective unilateral mastectomy with sentinel node biopsy due to breast cancer. The patient has a history of four episodes of anaphylaxis, one of which occurred during the induction of general anesthesia and resulted in cardiorespiratory arrest, which was reversed with advanced life support. The intradermal tests confirmed allergy to propofol, all types of neuromuscular relaxants, ketorolac, acetylsalicylic acid, generic antibiotics, and iodinated contrast agents. Following a multidisciplinary discussion that included anesthesiology, gynecology, allergy and clinical immunology, and the patient, it was decided to perform a thoracic epidural block in combination with interpectoral and pectoserratus plane blocks for the surgery. The patient requested anxiolysis via music therapy. The procedure was uneventful, and the patient was transferred to the post-anesthetic care unit, where she remained under vigilance for 12 hours.

## Introduction

Perioperative anaphylaxis is a challenging clinical situation. In most cases, it is caused by IgE-mediated immediate hypersensitivity. With an estimated incidence between 1:10,000 and 1:20,000 cases, it is associated with the use of drugs and substances contained in surgical and anesthesia materials [1]. These patients require a highly individualized anesthetic plan due to the limited availability of safe pharmacological options, the increased risk of perioperative anaphylaxis, and the need for close coordination with Immunoallergy teams. Antibiotics are the most commonly involved agents (48%), followed by neuromuscular relaxants (25%) [2]. Sugammadex is also a potential cause of anaphylaxis in one out of 2,500 cases [3]. For a favorable prognosis, it is imperative to recognize the signs and symptoms of anaphylaxis as early as possible. Anaphylaxis may present with a range of symptoms, most commonly hypotension, compensatory tachycardia, and bronchospasm (wheezing), while cutaneous signs such as rash or angioedema may be absent. As surgical drapes may delay the identification of some of them, it is important to maintain a high index of suspicion in cases of persistent hypotension resistant to vasopressor agents, even in the absence of skin lesions. Administration of adrenaline is the first-line treatment in the perioperative setting [4]. In order to confirm the diagnosis, serum levels of histamine and tryptase should be measured 30 minutes to two hours after the event and 24 hours later. The definitive identification of the causative agent is performed through intradermal testing [5].

In most cases, breast cancer surgery is done under general anesthesia. Regional techniques are often added to help manage postoperative pain and to reduce the need for opioids [6]. The most commonly used approaches include the paravertebral block (PV) and interpectoral and pectoserratus planes, which aim to cover the main sensory nerves of the breast and anterior chest wall [7]. Using regional anesthesia as the sole anesthetic technique for breast surgery is not routine and is usually considered only in specific situations, particularly when general anesthesia carries additional risk [8].

## Case presentation

The present clinical case describes a 55-year-old female patient, with the American Society of Anesthesiology (ASA) Physical Status Classification IV, proposed for an unilateral mastectomy with sentinel node biopsy to treat breast cancer. The patient has a history of previous episodes of anaphylaxis (Table 1), one of which occurred after general anesthesia induction and resulted in cardiorespiratory arrest that was reversed with advanced life support.

**Table 1:** Past episodes of anaphylaxis

Date	Context	Culprit	Symptoms
1978	Excretory urography	Iodinated contrast agent	Urticaria and severe hypotension
1982	Induction of general anesthesia for an appendectomy	Induction and neuromuscular blocking drugs	Hypotension and cardiorespiratory arrest (20 minutes of advanced life support)
1997	First intake of a folic acid capsule	Folic acid	Urticaria, facial and limb angioedema, and hypotension
2007	Intake of 1 gram of acetylsalicylic acid	Acetylsalicylic acid	Hypotension

In this context, the patient began follow-up at the Allergy and Clinical Immunology Department and was diagnosed with hypersensitivity to nickel, sulfites, and contrast agents. She was also diagnosed with chronic urticaria, which worsens with histamine-releasing foods, preservatives, and additives. She is also under evaluation for a mast cell clonal disorder. Her baseline tryptase levels are normal, and the D816V mutation of the c-KIT gene, involved in the vast majority of patients with systemic mastocytosis, has been ruled out [9]*.* A more in-depth study has not yet been conducted. Given the time-sensitive nature of the surgery, it was decided not to wait for this study. Other medical history includes Hashimoto's thyroiditis and migraines.

Allergy testing confirmed that the patient has hypersensitivity to neuromuscular blocking agents from different pharmacological classes. No reactions were identified to midazolam, bupivacaine, or penicillins (Table 2). She also underwent a basophil activation test, which was negative for fentanyl and thiopental, but came back positive for propofol and cisatracurium (Table 3).

**Table 2:** Intradermal tests

Neuromuscular blocking agents			
Drug	Concentration	Dilution	Result
Succinylcholine	20 mg/mL	1/1000	10 mm wheal, erythema and pruritus
Atracurium	1 mg/mL	1/1000	12 mm wheal, erythema and pruritus
Vecuronium	4 mg/mL	1/1000	8 mm wheal, erythema and pruritus
Rocuronium	10 mg/mL	1/1000	8 mm wheal, erythema and pruritus
30 minutes after the patient presented with tachycardia, hypotension, and glottic edema			
Local anesthetics and benzodiazepines			
Drug	Concentration	Dilution	Result
Bupivacaine	2.5 mg/mL	1/100 and 1/10	Negative
Levobupivacaine	2.5 mg/mL	1/100 and 1/10	Negative
Midazolam	5 mg/mL	1/1000, 1/100 and 1/10	Negative
Penicillins			
Drug	Concentration	Dilution	Result
Penicilloyl-polylysine (PPL)	5.0 × 10^5^ mol/L	1/1	Negative
Minor determinants (MD)	2.0 × 10^2^ mol/L	1/1	Negative

**Table 3:** Basophil activation test

Drug	Result
Fentanyl	Negative
Thiopental	Negative
Propofol	Positive
Cisatracurium	Positive

In the past, she had tolerated dental procedures under local anesthesia and a hemorrhoidectomy under spinal anesthesia without complications.

Given the severity of the anaphylactic reaction, the Allergy and Clinical Immunology team advised that, in future procedures, halogenated inhalational agents and locoregional techniques should be prioritized whenever possible, in order to reduce the risk of further reactions.

The anesthetic plan was made after a multidisciplinary discussion with involvement of an immunoallergologist, a gynecologist, and two senior anesthesiologists. Following discussion with the patient about the possible anesthetic options, and taking into account the potential risks and benefits of each, it was decided to perform a thoracic epidural block in combination with an interpectoral and pectoserratus plane blocks for the surgery. The patient was offered the option of anxiolysis with midazolam for the procedure, but she preferred non-pharmacological methods, namely, music through headphones.

Emergency drugs and equipment were prepared beforehand, and standard ASA monitoring was started, following which 2 mg of clemastine and 125 mg of methylprednisolone were administered. As decided by the team, Augmentim® (amoxicillin and clavulanic acid) was administered orally as a prophylactic surgical antibiotic.

The epidural catheter insertion at the T4-T5 level proceeded without complications. A total of 6 mL of 0.5% levobupivacaine was slowly titrated until block installation. The block was tested with loss of thermal sensitivity to alcohol, and a sensory level between T1 and T9 was observed. Under ultrasound visualization, a pectoserratus plane block was performed with the administration of 20 mL of 0.5% levobupivacaine, followed by the interpectoral plane block, with the administration of 10 mL of the same solution. The patient listened to her selected music via headphones throughout the surgery.

The procedure lasted 135 minutes. Spontaneous ventilation and hemodynamic stability were maintained without the need for vasopressor administration (Table 4).

**Table 4:** Perioperative parameters

Time	Systolic arterial pressure	Diastolic arterial pressure	Heart rate	Peripheral oxygen saturation
00:00 h	125 mmHg	79 mmHg	72 bpm	96%
00:30 h	118 mmHg	80 mmHg	65 bpm	97%
01:00 h	130 mmHg	70 mmHg	58 bpm	96%
01:30 h	125 mmHg	65 mmHg	58 bpm	98%
02:00 h	122 mmHg	58 mmHg	57 bpm	98%
02:30 h	124 mmHg	60 mmHg	58 bpm	98%

After two hours of surgery, an additional bolus of 2 mL of 0.5% levobupivacaine was administered. Following the procedure, the patient was transferred to the Post-Anesthetic Care Unit (PACU), where she remained under vigilance for 12 hours.

For postoperative pain control, the patient had access to patient-controlled epidural analgesia (PCEA), set to deliver 4 mL boluses of 0.15% levobupivacaine on demand with a 20-minute lockout between doses. During her stay in the PACU, she required only two boluses. Additionally, Brufen® (ibuprofen) and Ben-u-Ron® (acetaminophen) were available as the patient had a history of taking them without any adverse reactions. However, she opted to rely only on PCEA, meditation, and music therapy as they were sufficient.

## Discussion

Currently, a common anesthetic management for breast cancer surgery involves combining general anesthesia with regional anesthesia. There are several case reports in the literature that showed a successful management of breast cancer surgery under regional anesthesia (PV block, interpectoral and pectoserratus plane block, superficial cervical plexus block, and others). These can be viable alternatives to general anesthesia and could potentially mitigate risks in patients with comorbidities. Additionally, the mainstay of regional anesthesia is to provide an effective postoperative opioid sparing analgesia and a reduction in postoperative nausea and vomiting. Table 5 describes clinical cases using regional techniques for breast surgery anesthesia described in the literature.

**Table 5:** Clinical cases using regional techniques for breast surgery anesthesia described in the literature Sources: Refs [10-13]

Clinical case	Regional technique used	Comments
1 - Young female patient with interatrial communication and severe pulmonary hypertension proposed for modified radical mastectomy [10].	Anesthetic paravertebral block + pectoserratus planeblock + superficial cervical plexus block	Successful
2 - 66-year-old female patient with history of severe aortic stenosis, atrial fibrillation, hypertension, diabetes proposed for radical mastectomy [11].	Anesthetic thoracic paravertebral block + subclavicular brachial plexus block + interpectoral plane block	Successful
3 - 77-year-old male patient with history of myocardial infarction with dilated cardiomyopathy and severe impairment of ejection fraction proposed for radical mastectomy and axillary clearance [12].	Anesthetic erector spinae plane block + selective brachial plexus block	Successful
4 - Excision of a huge breast fibroadenoma under regional anesthesia [13].	Anesthetic pectoserratus plane block + internal intercostal plane block	Successful. The author also emphasizes regional blocks as an alternative to general anesthesia because there is no need to control visceral pain in breast and anterior thoracic wall surgeries, as opposed to abdominal surgery.

In our case, the primary option for regional anesthesia allowed the total avoidance of known allergenics. Although some agents (e.g., fentanyl and midazolam) tested negative, others commonly used in general anesthesia - including propofol and multiple neuromuscular blockers - elicited positive results. Given the history of severe anaphylaxis, including cardiorespiratory arrest, the team opted for avoiding systemic drugs unless absolutely necessary. Regional anesthesia offered complete surgical anesthesia and analgesia. The patient’s will was also considered in the decision-making process, which expressed a preference for remaining conscious during the procedure. Additionally, general anesthesia is associated with an increased risk of nausea and vomiting after breast cancer surgeries. Using regional anesthesia as a sole technique would potentially minimize its occurrence [14].

Alternative approach to this case was a general anestesia with supraglottic airway device placement using fentanyl and a pure inhalational induction with volatile anesthetics or an induction with fentanyl and midazolam, both combined with locoregional techniques.

To anticipate potential critical events during the case, such as severe hypotension, urticaria, glottic edema, or cardiopulmonary arrest, emergency drugs, including adrenaline, phenylephrine, hydrocortisone, and noradrenaline, were prepared and kept available in the operating room throughout the procedure. Additionally, airway management equipment for orotracheal intubation was readily available.

Local anesthetics (LA) are generally safe drugs with regard to allergic reactions corresponding to <1% of their adverse effects. Levobupivacaine, an aminoamide LA, has the lowest incidence of allergic reactions [15]. Preservatives (methylparaben) and antioxidants (sulfites) present in some LA preparations are responsible for some of the allergic reactions associated with these drugs [16].

To perform a mastectomy with sentinel lymph node biopsy under regional anesthesia, it is essential to understand the innervation of this anatomical region. The anterolateral region of the thorax and the axillary region are innervated by different nerve groups [15]. For sentinel lymph node biopsy axillary dissection, regional blocks, such as interpectoral and pectoserratus plane blocks, erector spinae plane (ESP) block, and PV block, have been described as successful anesthesia techniques. It should be noted, however, that none of these regional techniques can guarantee a complete block for all the surgical territories in this clinical case [17,18].

The association of the thoracic epidural block at the level of T4-T5 allowed the blockade of the corresponding intercostal nerves bilaterally, which innervate the skin and the underlying breast tissue and also the blockade of sympathetic ganglionic chain branches. It also made possible a safe postoperative analgesia using the same LA.

The breast and axillary regions are innervated by a complex network of nerves derived not only from thoracic spinal roots (T2-T6) but also from cervical nerve roots (C3-C4 and C5-C7). Blocking these cervical-origin nerves is essential in breast surgery, particularly in procedures involving the pectoral muscles and axillary dissection. Table 6 describes the nerves involved and their innervation area.

**Table 6:** Breast and axillary region innervation

Nerve	Root origin	Innervation area
Anterior and lateral cutaneous branches of the intercostal nerves	T2–T6	Breast parenchyma and skin [7]
Intercostobrachial nerve	T2	Axilla and medial upper arm [7]
Supraclavicular nerves	C3–C4	Superior pole of the breast and upper chest [7]
Pectoral nerves (medial and lateral)	C5–T1	Pectoralis major and minor muscles [7]
Long thoracic nerve	C5–C7	Serratus anterior muscle [8]
Thoracodorsal nerve	C6–C8	Latissimus dorsi muscle [8]

While a thoracic epidural can effectively block thoracic dermatomes and provide visceral and somatic analgesia in the T2-T6 distribution, it does not cover nerves of cervical origin, such as the supraclavicular and pectoral nerves. Furthermore, the intercostobrachial nerve has high anatomical variability and is not always reliably anesthetized by neuraxial techniques. We associated the interpectoral and pectoserratus plane blocks to guarantee the blockade of the pectoral nerves and the long thoracic nerve, thus obtaining effective anesthesia in all regions involved in the surgical procedure [6].

Thoracic PV block was also described as an effective anesthetic technique in breast surgery, and it was also considered for this case. However, the anesthetic reliability of the epidural block and the senior anesthesiologist’s experience with this technique favored this choice [6,19].

Another fundamental aspect was the multidisciplinary team planning and anticipation of possible critical events. A very important aspect was the patient’s understanding of her medical condition and her self-control by non-pharmacological methods (music and meditation) that were useful to enhance her well-being and reduce anxiety [20].

## Conclusions

The decision to proceed with breast cancer surgery using locoregional techniques without any sedation or general anesthesia was carefully planned due to the patient’s severe allergic history, and her involvement in the decision-making process regarding the anesthesia technique was crucial. Regional anesthesia provided good surgical conditions and effective intraoperative and postoperative analgesia and mitigated risks related to general anesthesia. At the end of the procedure, the patient expressed overall satisfaction with the entire process.

The use of thoracic epidural anesthesia in association with interpectoral and pectoserratus plane blocks highlights their efficacy and safety, reinforcing their role as a viable alternative in oncologic breast surgery involving a sentinel lymph node biopsy. We highlight that this approach can serve as a primary anesthetic strategy in selected high-risk patients and remind of the value of individualized multidisciplinary collaboration.
